# Prediction of Monosodium Urate Crystal Deposits in the First Metatarsophalangeal Joint Using a Decision Tree Model

**DOI:** 10.2174/0115734056355443250505051813

**Published:** 2025-05-22

**Authors:** Jiachun Zhuang, Lin Liu, Yingyi Zhu, Yunyan Zi, Hongjing Leng, Bei Weng, Lina Chen, Haijun Wu

**Affiliations:** 1 Guangdong Cardiovascular Institute, Guangdong Provincial People’s Hospital, Guangdong Academy of Medical Sciences, Guangzhou, 510080, Guangdong, P.R China; 2 Department of Radiology, Guangdong Provincial People’s Hospital, Guangzhou 510080, Guangdong, P.R. China; 3 Department of Radiology, Huizhou First Hospital, Huizhou, 516001, Guangdong, P.R. China; 4 Department of Medical Imaging, Fuwai Yunnan Hospital, Chinese Academy of Medical Sciences, Kunming 650102, Yunnan, P.R. China; 5 The First Affiliated Hospital of Sun Yat-sen University, Guangzhou, 510080, Guangdong, P.R. China; 6 CT Collaboration, Siemens Healthcare Ltd, Guangzhou 510080, Guangdong, P.R. China

**Keywords:** Crystal arthropathies, gout, Metatarsophalangeal, Decision tree model

## Abstract

**Background:**

Despite the increasing prevalence of hyperuricemia and gout, there remains a relative paucity of research focused on the use of straightforward clinical and laboratory markers to predict urate crystal formation. The identification of such predictive markers is crucial, as they would greatly enhance the ability of clinicians to make timely and accurate diagnoses, leading to more effective and targeted therapeutic interventions.

**Objective:**

The aim of this study was to evaluate the diagnostic value of various easily obtainable clinical and laboratory indicators and to establish a decision tree (DT) model to analyze their predictive significance for monosodium urate (MSU) deposition in the first metatarsophalangeal (MTP) joint.

**Methods:**

A retrospective study was conducted on 317 patients who presented to the outpatient clinic with a gout flare between January 2023 and June 2024 (181 cases with MSU deposition in the first MTP joint and 136 cases without such deposition). Clinical and laboratory indicators included gender, age, disease course, serum uric acid (SUA), glomerular filtration rate (GFR), serum creatinine (SCR), C-reactive protein (CRP), and erythrocyte sedimentation rate (ESR). Statistical analysis methods, including T-test, logistic regression and decision tree, were used to analyze the predictors of MSU deposition in the first MTP joint. The performance of the DT model was evaluated using receiver operating characteristic (ROC) curves and a 5-fold cross-validation method was used to ensure the robustness of the study results.

**Results:**

Disease course, GFR, SUA, age, and SCR emerged as significant predictors of MSU deposition in the first MTP joint in both LR and DT analyses. The DT model exhibited superior diagnostic performance compared to the LR model, with a sensitivity of 83.4% (151/181), specificity of 56.6% (77/136), and overall accuracy of 71.9% (228/317). The importance of predictive variables in the DT model showed disease course, GFR, SUA, age, and SCR as 53.36%, 21.51%, 15.1%, 5.5% and 4.53%, respectively. The area under the ROC curve predicted by the DT model was 0.752 (95% CI: 0.700~0.800).

**Conclusion:**

The DT model demonstrates strong predictive capability. Disease duration, GFR, SUA, age, and SCR are pivotal factors for predicting MSU deposition at the first MTP joint, with disease course being the most critical factor.

## 1 INTRODUCTION

Gout is a prevalent chronic inflammatory condition characterized primarily by the deposition of monosodium urate (MSU) crystals [1-3]. These crystals are typically found in the joints or tendons of the feet or ankles, particularly in the first metatarsophalangeal (MTP) joint [1, 3]. The accumulation of MSU crystals is considered the principal mechanism behind the bone erosion and structural joint damage associated with gout [4, 5]. Therefore, the identification of MSU crystal deposition is crucial for the diagnosis and treatment of gout patients [6, 7].

The EULAR recommends a three-step approach for diagnosing gout [8]. The first step relies on MSU crystal identification in synovial fluid or tophus aspirates. If this is not feasible, the second step relies on clinical diagnosis based on the presence of hyperuricemia and clinical features of gout. The final step suggests that, in cases where the clinical diagnosis of gout is uncertain and crystals cannot be identified, imaging studies, especially ultrasound (US) or dual-energy computed tomography (DECT), should be conducted to find radiological evidence of MSU crystal deposition.

The typical first presentation of gout is an intensely painful acute inflammatory arthritis (gout flare) affecting a lower limb joint [1, 9]. In the scenario where a patient presents to the outpatient clinic for the first time with a gout flare, it is deemed inappropriate to hastily proceed with invasive procedures such as synovial fluid aspiration. Typically, adhering to clinical guidelines, our physicians would opt for a blood test to assess the levels of serum uric acid (SUA) to determine the presence of hyperuricemia [10]. Furthermore, additional common indicators such as serum creatinine (SCR), estimated glomerular filtration rate (eGFR), C-reactive protein (CRP), and erythrocyte sedimentation rate (ESR) are often utilized, and these test results are usually readily accessible. Despite these assessments, the results of blood tests, whether indicating hyperuricemia or not, do not provide clinicians with a definitive diagnosis of gout or confirmation of tophi deposition in the patient [11]. The final recommendation in the guidelines is to perform imaging studies. However, the indiscriminate use of imaging modalities like dual-energy computed tomography (DECT) in certain cases may lead to patient discontent and diminish the physician's engagement in the diagnostic process, potentially undermining the perceived authority of the physician's diagnostic acumen [12].

When should imaging studies such as DECT be employed? This question arises as we consider the clinical scenario where a physician, equipped with the patient's complaints and the results of blood tests, seeks a conclusive determination of whether the patient has tophi deposits. Our research aims to develop a model that can provide a clear directive on the presence of gout crystal deposition using minimal clinical data. This model will incorporate easily accessible clinical indicators such as the patient's age, the duration of symptoms since the first episode of gout-related symptoms, and laboratory results of gout-related markers such as serum uric acid (SUA), glomerular filtration rate (GFR) and serum creatinine (SCR). The model will focus on the first MTP joint as an example, given its commonality as the initial site of presentation in gout cases. Utilizing this model enables clinicians to make a preliminary estimation of whether patients with suspected gout have MSU deposits in the first MTP joint during their initial visit, thereby facilitating an accurate determination of whether to conduct a DECT examination of the foot.

## 2 MATERIALS AND METHODS

### 2.1 Patients

This retrospective study was approved by the Ethics Committee of Guangdong Provincial People's Hospital, and written informed consent was obtained from all participants. In this single-center study, we conducted a retrospective analysis of patients who presented at our outpatient clinic with their first gout flare between January 2023 and June 2024. The inclusion and exclusion criteria are detailed in Fig. (**1**). Ultimately, 181 patients were included in the study.

### 2.2 Clinical and Laboratory Indicators

In this study, disease course was defined as the time from the first gout flare to the current clinic visit. The laboratory parameters evaluated included SUA, SCR, GFR, ESR, and CRP. GFR was calculated based on creatine using the modified Modification of Diet in Renal Disease (MDRD) equation [c-aGFR(ml/min per1.73 m^2^) = 175× Pcr-1.234 × age-0.179 × 0.779 (if female)] [13].

### 2.3 Dual-energy CT Protocol

DECT images were retrieved with the commercial software Syngo.via (version VB 10B; Siemens Healthineers. MSU deposits were visualized and color-coded and volume measurements were performed automatically by the clinical software application Syngo Dual Energy Gout. The urate ratio was set to 1.36 and the smoothing range at 4. Fluid was set to a minimum of 150 Hounsfield units (HU) for the 80kV/ Sn140kV images. The nail bed, skin, submillimeter, motion and beam hardening artefacts were excluded from the analysis and volume measurement [14]. The images are collectively evaluated by a junior radiologist with two years of diagnostic experience and a senior radiologist with over two decades of diagnostic expertise. Their analysis of the DECT image to determine the presence or absence of MSU deposits in the first MTP joint demonstrated excellent Inter-reader agreement (Kendall’s W=0.934, p<0.001), with a high level of reproducibility indicated by a kappa value of 0.945.

### 2.4 Statistical Methods and Decision Tree Modeling

Statistical analyses via t-test, chi-square test, and logistic regression analysis were performed using SPSS version 28.00 (SPSS, Chicago, Illinosis, USA). A P-value of <0.05 was considered significant. For the decision tree model, we used the Decision Tree classifier in Python with the *pydotplus* package. The decision tree model determines the optimal cut-points of every feature from the most influential to the trivial, using an information criterion such as the Gini index. To avoid overfitting problems, we applied the pruning technique, which limits the number of cases in each terminal node and the depth of the hierarchical tree.

## 3 RESULTS

### 3.1 Participant Characteristics

This study enrolled 317 people classified as either the MSU-positive in the first MTP group (181 cases) or the MSU-negative in the first MTP group (136 cases). There were 294 males and 23 females, with a median age of 49 years. The median disease course was 7 years, with median levels of SUA at 8.99 mg/dL, GFR at 89.09 ml/min, SCR at 1.01, CRP at 10.95 mg/L, and ESR at 26 mm/h Table **1**.

On comparing clinical features, no significant differences in the presence of MSU deposition in the first MTP were observed between different groups regarding gender, CRP and ESR. However, there were significant differences in the presence of MSU deposition in the first MTP between different groups regarding age, disease course, SUA, GFR, and SCR (Table **1**).

### 3.2 Decision tree Analysis

Five significant predictors were used to construct a decision tree model of the risk of MSU deposition in the first MTP joint: GFR, disease course, age, SCR, and SUA, as shown in Fig. (**2**). To ensure robust model performance, we implemented hyperparameter optimization through a rigorous process involving 5-fold cross-validation and a grid search algorithm. The grid search explored a wide range of hyperparameter combinations, and the optimal combination was selected based on the highest cross-validation accuracy. Ultimately, the optimal hyperparameter configuration identified for the DT model was as follows: 'criterion': 'gini', 'splitter': 'best', 'max_feat': 3, and 'max_depth': 3. The DT model was developed with a maximum depth of 3 levels, resulting in a total of 14 nodes, which includes 8 terminal nodes. Each node in the tree represents the probability of MSU deposition corresponding to specific branches. The max_depth parameter was explicitly set to 3 to constrain the tree's complexity. This shallow depth ensures that the model remains interpretable and avoids overfitting the training data. Additionally, the grid search implicitly performed a form of pre-pruning by evaluating and selecting models with optimal depth and feature constraints. The optimized DT model achieved a strong balance between accuracy and interpretability.

**Fig. (2):**
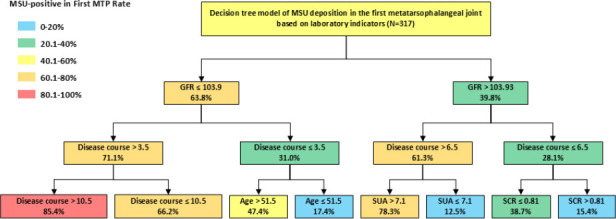
Decision tree model of the risk for MSU deposition in the first MTP.

The results showed that the probability of MSU deposits in the first MTP in patients with a GFR ≤ 103.93 is 63.8% (229 cases), which is higher than that in patients with a GFR > 103.93 (39.8%; 88 cases). In patients with a GFR ≤103.93, the probability of MSU deposits in the first MTP among patients with a disease course > 3.5 years is 71.1% (187 cases), which is higher than that in patients with a disease course ≤ 3.5 years (31.0%; 42 cases). Among patients with a GFR ≤ 103.93 and a disease course > 3.5 years, those with a disease course >10.5 years have a probability of 85.4% (48 cases) for MSU deposits in the first MTP, which is higher than that in patients with a disease course ≤10.5 (66.2%; 139 cases). Among patients with a GFR ≤ 103.93 and a disease duration ≤ 3.5 years, those with age> 51.5 have a probability of 47.4% (19 cases) for MSU deposits in the first MTP, which is higher than that in patients with age ≤51.5 (17.4%; 23 cases). In patients with a GFR >103.93, the probability of MSU deposits in the first MTP among patients with a disease course > 6.5 is 61.3% (31 cases), which is higher than that in patients with a disease course ≤ 6.5 years (28.1%; 57 cases). Among patients with a GFR > 103.93 and a disease course > 6.5, those with a SUA > 7.1 have a probability of 78.3% (23 cases) for MSU deposits in the first MTP, which is higher than that in patients with a SUA ≤ 7.1 (12.5%; 8 cases). In addition, among patients with a GFR > 103.93 and a disease course ≤ 6.5, those with an SCR ≤ 0.81 have a probability of 38.7% (31 cases) for MSU deposits in the first MTP, which is higher than that in patients with an SCR > 0.81 (15.4%; 26 cases) (Fig. **2**).

The importance of each node prediction variable to the construction of the model was different in the decision tree model, while the importance of a disease course, GFR, SUA, age and SCR were 53.36%, 21.51%, 15.1%, 5.5% and 4.53%, respectively Fig. (**3**).

### 3.3 Comparison Between the Decision Tree Model and Logistic Regression Model

To examine the performance of the decision tree model in predicting MSU deposits in the first MTP, we compared it to the conventional logistic regression model. The LR model included risk indicators that all appeared in the DT model-that is, the disease course, age, GFR, SCR and SUA. The comparison of the performance parameters of the DT and LR models is presented in Table **2**. The accuracy for the DT model was 71.9%, the sensitivity was 83.4%, and the specificity was 56.6%. However, the accuracy, sensitivity and specificity of the LR model were 68.8%, 81.2% and 52.2%, respectively (Table **2**). The DT model had better classification accuracy, sensitivity and specificity than the LR model. The area under the receiver operating characteristics (ROCs) curves (AUC) for the DT and LR models are 0.752 (0.700,0.800) and 0.718(0.660,0.772), respectively.

The performance of the DT model was evaluated using receiver operating characteristic (ROC) curves and a 5-fold cross-validation method was used to ensure the robustness of the study results. The results are graphically represented in the accompanying Fig. (**4**). It is noteworthy that the AUC values for both the training and validation groups of the decision tree model are closely aligned, suggesting that the model has maintained its predictive accuracy across different data splits. This consistency is a testament to the model's reliability and generalizability.

## 4 DISCUSSION

Identifying MSU deposition is crucial in the diagnosis and treatment of gout. The first MTP joint is a common initial site for urate deposition [9, 15]. In this study, we have developed a decision tree model based on limited clinical and laboratory information to predict MSU deposition in the first MTP joint, guiding clinicians in their judgment. When the predictive model yields a high-risk value for MSU deposition in the first MTP joint, we strongly recommend that clinicians order a DECT scan of the foot for a definitive diagnosis.

The disease course is the most significant predictor in our model. Since the patients we screened had not previously taken any urate-lowering medications, a longer disease duration in this context leads to an exponentially increased risk of gout. This makes disease duration the most crucial indicator for predicting urate crystal deposition in the first metatarsophalangeal joint. Furthermore, our findings align with those of Tam *et al*., who reported that MSU deposition in the first MTP joint is associated with both disease duration and flare-ups [16].

The first node in our model is GFR, rather than the traditionally considered SUA. Although the association between GFR and gout may be mediated through its influence on SUA levels-given that, under normal conditions, renal filtration plays a crucial role in SUA excretion-GFR holds greater importance in our predictive model [17-19]. This may be because GFR, as an indicator of renal function, is typically more stable, whereas SUA levels are influenced by various factors, such as diet and short-term physiological changes, which can lead to significant fluctuations in SUA levels [20].

The SUA value accounts for 15.1% of the importance in our model. A previous study by Pascart *et al*. demonstrated no statistically significant correlation between monosodium urate (MSU) crystal volume and SUA levels [21]. Furthermore, an observational DECT study documented only a weak correlation between MSU deposition and SUA levels [22]. Nonetheless, the SUA value still exhibits a certain role in our model. It is worth mentioning that the node value for SUA in the model is 7.1, which is very close to the standard hyperuricemia threshold (SUA > 7.0 mg/dL). This suggests a potential association between hyperuricemia and MSU crystal formation. Additionally, this proximity enhances the interpretability of our model in clinical practice.

In our model, age is an included predictive factor with a threshold of 51.5. This may be attributed to the fact that as age increases, metabolic capacity diminishes, and the risk of MSU deposition in the first MTP joint also escalates with age. Studies have indicated that gout typically manifests in middle age, peaking particularly around the age of 50 [23]. The age threshold in our model closely aligns with this finding, suggesting that our model is of high quality and exhibits favorable trends, capable of yielding unexpected clinical outcomes during its application.

SCR shows lower importance in our model. This may be related to the fact that SCR is not highly sensitive to the early detection of renal dysfunction [24]. Under normal conditions, the production rate of SCR is relatively constant, but its levels can also be affected by factors such as muscle mass, dietary habits, and physical activity [25]. When SCR levels begin to rise, it may indicate that renal function has already suffered some degree of impairment. This potential to reflect renal function damage contributes to its value in predicting the progression of gout and may explain why it serves as a potential predictor for SUA deposition in the first MTP joint.

In summary, the DT model achieved an accuracy of 71.9%, a sensitivity of 83.4%, and a specificity of 56.6%. In contrast, the LR model recorded an accuracy of 68.8%, a sensitivity of 81.2%, and a specificity of 52.2% (Table **2**). Overall, the DT model outperformed the LR model in terms of classification accuracy, sensitivity, and specificity. The DT model demonstrates potential clinical value in predicting MSU deposition in patients with first MTP joint involvement. Although the specificity of the DT model is relatively low, this may be attributed to a greater emphasis on enhancing accuracy and sensitivity during the optimization process. In clinical practice, when combined with straightforward patient history and laboratory test results, the DT model can serve as an initial screening tool for the early identification of high-risk patients. The critical aspect of early screening lies in maximizing sensitivity to reduce the rate of false negatives, ensuring that more patients receive timely diagnoses. Future research could further optimize model performance, particularly with respect to increasing specificity, by incorporating additional features or utilizing ensemble learning methods.

This study has several limitations that should be acknowledged. First, as a single-center retrospective study, our analysis may be subject to selection bias, data incompleteness, and variations in diagnostic or recording practices over time. These factors could affect the reliability and generalizability of our findings. Second, our study focuses exclusively on MSU deposition in the first MTP joint, which, although typically the earliest site of MSU deposition, limits the comprehensiveness of our results. The findings may not fully capture the patterns or predictive factors of MSU deposition in other joints or throughout the body. Third, the detection of MSU deposition in early gout poses a significant challenge. In patients with early disease, urate may be deposited in joint fluid or form liquid tophi, both of which are difficult to detect using DECT due to the low density of crystals within a voxel [26]. This limitation could lead to underdiagnosis or misclassification of early cases, potentially affecting the model's performance and clinical applicability. Future studies should consider multi-center, prospective designs with larger and more diverse cohorts to validate and extend these results. Additionally, incorporating advanced imaging techniques or machine learning methods to improve the detection of early MSU deposition could enhance the model's predictive accuracy and clinical utility.

## CONCLUSION

In conclusion, this study demonstrates that the MSU deposition in the first MTP joint of patients can be predicted using five clinically accessible factors: disease course, GFR, SUA, age, and SCR, with disease course being the most significant predictive factor. The decision tree model derived from our analysis is of high quality, with high accuracy, sensitivity, and specificity, and has interpretable thresholds that are easy to use in clinical practice. We recommend that patients with a high risk of MSU deposition in the first MTP joint, as indicated by the decision tree model, undergo a DECT scan of the foot for further evaluation. Specifically, in the context of early screening, we recommend that patients with a predicted probability of MSU deposition between 60% and 100% undergo DECT of the foot for further evaluation. For patients with a predicted probability between 40% and 60%, we suggest considering DECT imaging on a conditional basis, taking into account factors such as availability and cost-effectiveness.

## Figures and Tables

**Fig. (1):**
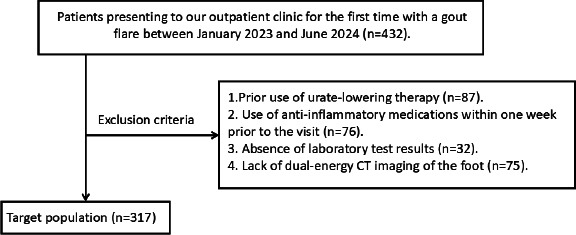
A flow chart for study population selection.

**Fig. (3):**
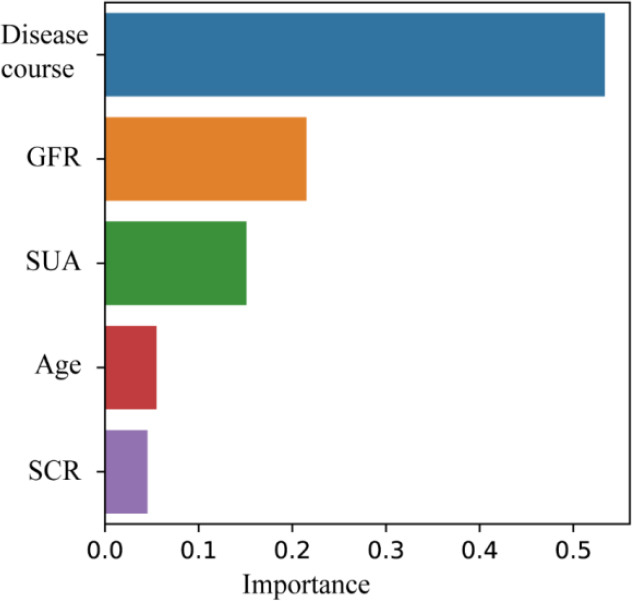
Importance of predictive variables in decision tree model. This figure shows the importance of each influence factor to the result prediction in the decision tree model. As a root node variable, disease course is the most important predictive variable.

**Fig. (4):**
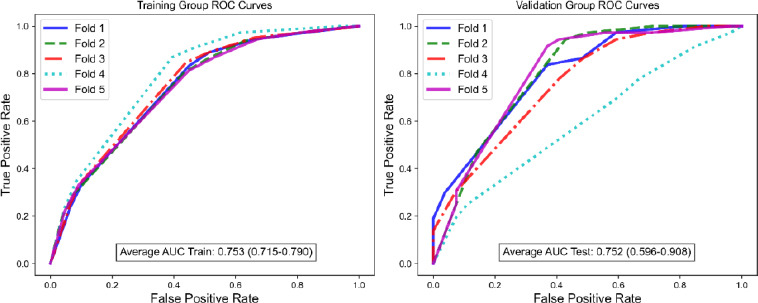
Receiver operating characteristic (ROC) curve of the decision tree (DT) model with a five-fold cross-validation scheme.

**Table 1:** Characteristics of 317 patients stratified by presence of MSU deposition in first MTP joint.

**Characteristics**	**Total (n=317)**	**MSU-negative in First MTP (n=136)**	**MSU-positive in First MTP (n=181)**	***p***
Gender	-	-	-	-
Male	294 (92.7%)	124 (91.2%)	170 (93.9%)	.475
Female	23 (7.3%)	12 (8.8%)	11 (6.1%)	-
Age (y)	49.00 (38, 62)	46.00 (33.00, 55.50)	53.00 (40.00, 65.00)	**<.001**
Disease course (y)	7.00 (4.00, 10.00)	5.00 (2.50, 10.00)	9.00 (5.00, 11.00)	**<.001**
SUA (mg/dL)	8.99 (7.26, 10.70)	8.78 (6.96, 10.29)	9.44 (7.45, 11.00)	**.030**
GFR (ml/min)	89.09 (69.28, 106.36)	92.49 (76.11, 115.22)	83.82 (62.56, 98.61)	**<.001**
SCR (mg/dL)	1.01 (0.87, 1.2)	0.99 (0.82, 1.13)	1.02 (0.91, 1.31)	**.002**
CRP (mg/L)	10.95 (3.57, 40.30)	11.70 (3.50, 31.50)	10.60 (3.73, 44.67)	.990
ESR (mm/h)	26.00 (10.00, 57.00)	27.50 (10.00, 64.50)	23.00 (10.00, 55.00)	.555

Unless gender data, data are median (IQR). Statistical analysis was performed with t test. Gender data are quantity with the percentage in parentheses. Statistical analysis was performed with the Pearson 's chi-square test. MSU= monosodium urate, MTP= metatarsophalangeal joint, SUA= serum urate, GFR= glomerular filtration rate, SCR= serum creatinine, CRP=C-reactive protein, ESR=erythrocyte sedimentation rate. * Significant value. p value<0.05 indicates statistical significance.

**Table 2:** Comparison of the performance parameters of the logistic regression and decision tree models.

	**Decision Tree Model**		**Logistic Regression Model**	
	**Predicted positives**	**Predicted negatives**	**Predicted positives**	**Predicted negatives**
Diagnosed positives	151 (TP)	30 (FN)	147 (TP)	34 (FN)
Diagnosed negatives	59 (FP)	77 (TN)	65 (FP)	71 (TN)
Accuracy	0.719		0.688	
Sensitivity	0.834		0.812	
Specificity	0.566		0.522	
AUC (95% CI)	0.752 (0.700-0.800)		0.718 (0.660-0.772)	
Cutoff	0.662		0.474	

TP true positive, TN true negative, FP false positive, FN false negative. a Accuracy=(TP+TN)/ (TP+FP+TN+FN). b Sensitivity = TP/(TP+FN). c Specifcity = TN/(TN+FP).

## Data Availability

The data supporting the findings of the article is available within the article.

## LIST OF ABBREVIATIONS

DECT: Dual-energy computed tomography
MSU: Monosodium urate
MTP: Metatarsophalangeal joint
SUA: Serum urate
GFR: Glomerular filtration rate
SCR: Serum creatinine
CRP: C-reactive protein
ESR: Erythrocyte sedimentation rate
DT: Decision tree model
LR: Logistic regression model
ROC: Receiver operating characteristic

## STANDARDS OF REPORTING

STROBE guidelines were followed.
